# Proteomics analysis in rats reveals convergent mechanisms between major depressive disorder and dietary zinc deficiency

**DOI:** 10.1007/s43440-024-00681-7

**Published:** 2024-12-03

**Authors:** Łukasz Gąsior, Bartłomiej Pochwat, Monika Zaręba-Kozioł, Jakub Włodarczyk, Andreas Martin Grabrucker, Bernadeta Szewczyk

**Affiliations:** 1 Maj Institute of Pharmacology, Department of Neurobiology, Polish Academy of Sciences, Smętna 12, Kraków, 31-343 Poland; 2 Laboratory of Cell Biophysics, Nencki Institute of Experimental Biology, Polish Academy of Sciences, Ludwika Pasteura 3, Warsaw, 02-093 Poland; 3 Dept. of Biological Sciences, University of Limerick, Limerick, V94PH61 Ireland; 4 Bernal Institute, University of Limerick, Limerick, V94PH61 Ireland; 5 Health Research Institute (HRI), University of Limerick, Limerick, V94PH61 Ireland

**Keywords:** Depression, Zinc deficiency, Mitochondrial dysfunction, Proteomic analysis

## Abstract

**Background:**

Preclinical and clinical studies have shown that dietary zinc deficiency can lead to symptoms similar to those observed in major depressive disorder (MDD). However, the underlying molecular mechanisms remain unclear. To investigate these mechanisms, we examined proteomic changes in the prefrontal cortex (PFC) and hippocampus (HP) of rats, two critical brain regions implicated in the pathophysiology of depression.

**Methods:**

Rats were fed diets either adequate in zinc (ZnA, 50 mg Zn/kg) or deficient in zinc (ZnD, <3 mg/kg) for four weeks. High-throughput proteomic analysis was used to detect changes in protein expression, supplemented by enzyme activity assay for mitochondrial complexes I and IV, examining their functional impacts.

**Results:**

ZnD led to significant alterations in protein expression related to zinc transport and mitochondrial function. Proteomic analysis revealed changes in zinc transporter family members such as Slc30a1 (6.64 log2FC), Slc30a3 (-2.32 log2FC), Slc30a4 (2.87 log2FC), Slc30a5 (5.90 log2FC), Slc30a6 (1.50 log2FC), and Slc30a7 (2.17 log2FC) in the PFC, and Slc30a3 (-1.02 log2FC), Slc30a5 (-1.04 log2FC), and Slc30a7 (1.08 log2FC) in the HP of rats subjected to ZnD. Furthermore, ZnD significantly affected essential mitochondrial activity proteins, including Atp5pb (3.25 log2FC), Cox2 (2.28 log2FC), Atp5me (2.04 log2FC), Cyc1 (2.30 log2FC), Cox4i1 (1.23 log2FC), Cox7c (1.63 log2FC), and Cisd1 (1.55 log2FC), with a pronounced decrease in complex I activity in the PFC.

**Conclusions:**

Our study demonstrates that ZnD leads to significant proteomic changes in the PFC and HP of rats. Specifically, ZnD alters the expression of zinc transporter proteins and proteins critical for mitochondrial function. The significant decrease in complex I activity in the PFC further underscores the impact of ZnD on mitochondrial function. These results highlight the molecular mechanisms by which ZnD can influence brain function and contribute to symptoms similar to those observed in depression.

**Supplementary Information:**

The online version contains supplementary material available at 10.1007/s43440-024-00681-7.

## Introduction

Major depressive disorder (MDD) is one of the most prevalent psychiatric disorders, characterized by the presence of sad, empty, or irritable moods and cognitive impairment [1]. MDD affects approximately 4.4% of the worldwide population, representing over 300 million people [2, 3]. The most well-known hypothesis explaining the onset of depression is the monoamine hypothesis, which posits that inadequate levels of neurotransmitters such as serotonin, norepinephrine, and dopamine lead to depressive symptoms [4]. Beyond the monoamine hypothesis, several other theories attempt to explain the onset and persistence of depression, including the neuroplasticity hypothesis [5, 6], inflammatory hypothesis [6], genetic and epigenetic [7], brain metabolic alterations, and mitochondrial dysfunctions [5, 6] and so-called micronutrient deficiency [7, 8]. The latter hypothesis points to impaired zinc homeostasis as a risk factor for depressive symptoms [9]. Indeed, considerable clinical data indicate that depressed patients exhibit significantly lower serum zinc levels than normal controls, which in some cases were found to be normalized after successful antidepressant therapy [10, 11]. One of the causes of reduced zinc levels in the body is an inadequate supply of this element in the diet. Clinical studies have shown a significant correlation between low dietary zinc supply and depressive symptoms [10, 12]. These observations set the stage for preclinical studies to establish a new animal model of depression based on experimentally induced zinc deficiency caused by a zinc-deficient diet [13–18]. Our laboratory has evaluated the effects of a zinc-poor diet (3 mg Zn/kg applied for 4 and 6 weeks) on the development of depressive behaviors in rats and determined the changes underlying these behaviors. We found that following 4 or 6 weeks of zinc restriction, rats exhibited behavioral despair, anhedonia, and reduced social behavior. We also measured the serum zinc concentration and found that both 4 and 6 weeks of a zinc-deficient diet significantly decreased the serum zinc level in rats [19]. Since a four-week zinc-poor diet produces almost the same effects as a six-week diet in subsequent studies, we limited our experiments to a four-week diet protocol [20, 21]. We also found that this experimentally induced zinc deficiency is associated with alterations in the N-methyl-D-aspartate receptor (NMDA) signaling pathways [19] and enhancement of oxidative/inflammatory parameters [21], which may be linked to depression-like behavior observed in rats.

Zinc is a crucial component of approximately 2,700 proteins within mammalian cells [22]. It plays critical roles in several biological processes, such as macromolecule synthesis, signal transduction, redox homeostasis, DNA synthesis, repair and methylation, and gene transcription [23–25]. Zinc is essential for neurotransmitter activity, neuroplasticity, and immune system response [26, 27]. Insufficient zinc intake is associated with increased plasma glucose levels, potentially contributing to metabolic syndrome and diabetic complications [28–30]. Given zinc’s extensive role, the mechanisms mentioned above underlying the depressive-like symptoms induced by ZnD may not be the only ones responsible for the pro-depressive changes. Therefore, we decided to characterize our model of depression further and conducted proteomic analysis in the brains of rats fed zinc-deficient feed for four weeks.

The PFC and the HP are critical for cognitive and behavioral regulation [31–34] and are key brain regions studied in mental health disorders such as depression and anxiety [31–34]. Zinc is notably concentrated in these brain regions, making them particularly sensitive to dietary zinc levels. Additionally, previous studies have shown that zinc deficiency differentially affects biochemical markers across brain regions, prompting us to conduct proteomic analyses of both PFC and HP [21]. Finally, our findings revealed substantial alterations in proteins associated with electron transport in the respiratory chain within the PFC but not HP. To confirm these results, we measured the activity levels of mitochondrial respiratory complexes I and IV in the PFC to validate these results.

## Materials and methods

### Animal housing

The Local Ethical Commission for Animal Experiments at Maj Institute of Pharmacology, Polish Academy of Sciences in Krakow approved all experiments (permit number 87/2016). Presented studies fulfilled the requirements of the EU Directive 2010/63/EU on protecting animals used for scientific purposes. Thirty-three male Sprague Dawley rats (Charles River, Germany), obtained at four weeks old, were housed in conventional plastic rodent cages (5–6 rats per cage) maintained at room temperature 22 ± 2 °C, 55 ± 10% humidity, and 12 h light/dark cycle, with lights on at 6:00 a.m), with ad libitum access to food and water. Rats were acclimatized for one week in a controlled lab environment with a standard diet containing 35 mg/kg of zinc.

### Diet regimen

Post-acclimatization (one week of handling), the rats were randomly divided into two groups. They were fed either a zinc-adequate diet (50 mg Zn/kg, control group, CTR) or a zinc-deficient diet (< 3 mg Zn/kg, ZnD), supplied by Altromin GmbH, Lage, Germany for four weeks, as described in our previous studies [19–21]. After four weeks on either a zinc-adequate or zinc-deficient diet, rats were euthanized through swift decapitation. Their brains were quickly removed and placed in a chilled 0.9% saline solution. PFC and HP were dissected, frozen on dry ice, and stored at -80 °C until further analysis.

### Mass spectrometry analysis

For protein isolation, tissue samples (*n* = 5 samples/group) were homogenized in a lysis buffer and diluted to a final protein concentration of 10 µg per aliquot in 50 mM ammonium bicarbonate buffer (pH 8.2) (Thermo Fisher Scientific, J60408.AP). The proteins were reduced by adding 10 mM dithiothreitol (DTT) (DTT-RO-Roche) and incubating the samples at 56 °C for 30 min to break disulfide bonds. Alkylation was then performed by adding 5 mM iodoacetamide (IAA) (Sigma Aldrich, I6125-5G) and incubating the samples in the dark at room temperature for 30 min to prevent reformation of disulfide bonds by modifying the cysteine residues.

After reduction and alkylation, the proteins underwent enzymatic digestion. Sequencing-grade trypsin from Promega (V511A) was added to the samples at a ratio of 1:50 (trypsin), and the digestion was carried out overnight at 37 °C. This step cleaved the proteins into peptides, making them more suitable for mass spectrometric analysis.

Peptide analysis was performed on a Q Exactive Orbitrap mass spectrometer (Thermo Fisher Scientific) interfaced with a Dionex UltiMate 3000 RSLC nanoLC system (Thermo Fisher Scientific) for liquid chromatography, which separated the peptides before mass spectrometric detection. Quality control checks of the mass spectrometer and the LC system were routinely conducted using standard peptide mixtures to ensure consistent performance and accuracy of the measurements.

The mass spectrometer was operated in data-dependent acquisition (DDA) mode. Peptides were ionized using a nano-electrospray ionization source, and the resulting ions were analyzed in the Orbitrap mass analyzer. Full MS scans were acquired at a resolution of 70,000 (at m/z 200) with a mass range of 300–2,000 m/z. The ten most intense precursor ions from each full scan were selected for fragmentation (MS/MS) using higher-energy collisional dissociation (HCD) with a normalized collision energy 28. Fragment ions were detected in the Orbitrap at a resolution of 17,500 (at m/z 200).

The raw mass spectrometric data were processed using MaxQuant software (version 1.5.3.30). Peptide identification was performed by searching the tandem mass spectra against the UniProt *Rattus norvegicus* database (number of sequences: 31,914, downloaded in April 2021) using the Andromeda search engine integrated into MaxQuant. The search parameters included an error tolerance of ± 0.01 Da for precursor ions and ± 0.02 Da for fragment ions. Carbamidomethylation of cysteine was set as a fixed modification, while methionine oxidation was considered as a variable modification.

Protein identification and quantification were also carried out in MaxQuant, with label-free quantification (LFQ) settings including a minimum ratio count of 2. The ‘Match between runs’ option was enabled to match peptide identifications across different samples and runs, improving the consistency and coverage of protein quantification. Protein groups were quantified based on the LFQ intensities, and statistical analysis was performed to identify differentially expressed proteins between the experimental groups.

To ensure the robustness of the quantification results, data quality assessment included checks for peptide and protein identification rates, peptide spectral matches, and consistency of LFQ intensities across replicate samples. Bioinformatics analyses, such as gene ontology (GO) enrichment and pathway analysis, were performed on the identified proteins to gain insights into the biological processes and pathways affected by the experimental conditions.

### Assessment of mitochondrial respiratory complex activities

The activities of mitochondrial respiratory complexes I and IV were evaluated using the Complex I Enzyme Activity Assay Kit and Complex IV Rodent Enzyme Activity Microplate Assay Kit (Catalog number: ab109911, Abcam, Cambridge, UK), respectively. Freshly isolated prefrontal cortex tissues (*n* = 11–12 samples/group) were homogenized, with proteins extracted and quantified using standard procedures. For each assay, 10 µg of protein per 200 µl volume was prepared, following the manufacturer’s protocols for sample preparation, detergent extraction, and protein solubilization to ensure optimal enzyme activity detection.

Complex activities were measured in a spectrophotometric assay by monitoring the oxidation of cytochrome c as a decrease in absorbance at 550 nm, indicative of complex IV activity. A similar approach was adapted for complex I. Assays were performed in duplicate for each sample, with enzymatic activity calculated as the initial rate of absorbance change normalized to the protein concentration.

### Statistical analysis

Data from mass spectrometry analysis were processed to ensure accuracy and statistical significance using ANOVA with Bonferroni correction for multiple comparisons and two-sample t-tests for differential protein expression. Enrichment analysis was conducted via Metascape, considering pathways with a q-value < 0.05 as significant. The q-value represents the false discovery rate (FDR)-adjusted *p*-value, minimizing the likelihood of false positives in multiple comparisons. Only proteins with a log2 fold change ≥ 1 or ≤ -1 and a *p*-value < 0.05 after Bonferroni correction were included in the enrichment analysis. Volcano plots were generated in Python using the Seaborn library, while bar plots were created with the Plotly library, providing an interactive and detailed visualization of the data. For mitochondrial protein analysis, the Mouse MitoCarta3.0 compendium was used as a reference to identify mitochondrial proteins within our dataset. MitoCarta3.0 is a comprehensive database that catalogs proteins with strong evidence of mitochondrial localization, providing a basis for selective analysis of mitochondrial proteins.

The normality of enzymatic activity data for mitochondrial complexes was assessed using the Shapiro-Wilk test. As the data for Complex I, were not normally distributed, the Mann-Whitney U test was used to evaluate statistical significance. For Complex IV, which followed a normal distribution, an unpaired t-test was applied. A *p*-value of < 0.05 was considered statistically significant in both cases.

## Results

### ZnD induces alterations in protein expression in the PFC and HP of rats

A comprehensive, high-throughput quantitative proteomic analysis revealed the impact of ZnD on the proteomic landscape of the PFC and HP. Our findings in the PFC identified 2,463 proteins that met our criteria, with a false discovery rate (FDR) of less than 1%. The differential analysis highlighted 86 proteins significantly altered by a 4-week ZnD, with 37 downregulated and 49 upregulated proteins, as detailed in Supplementary Table S1.

**Fig. 1:**
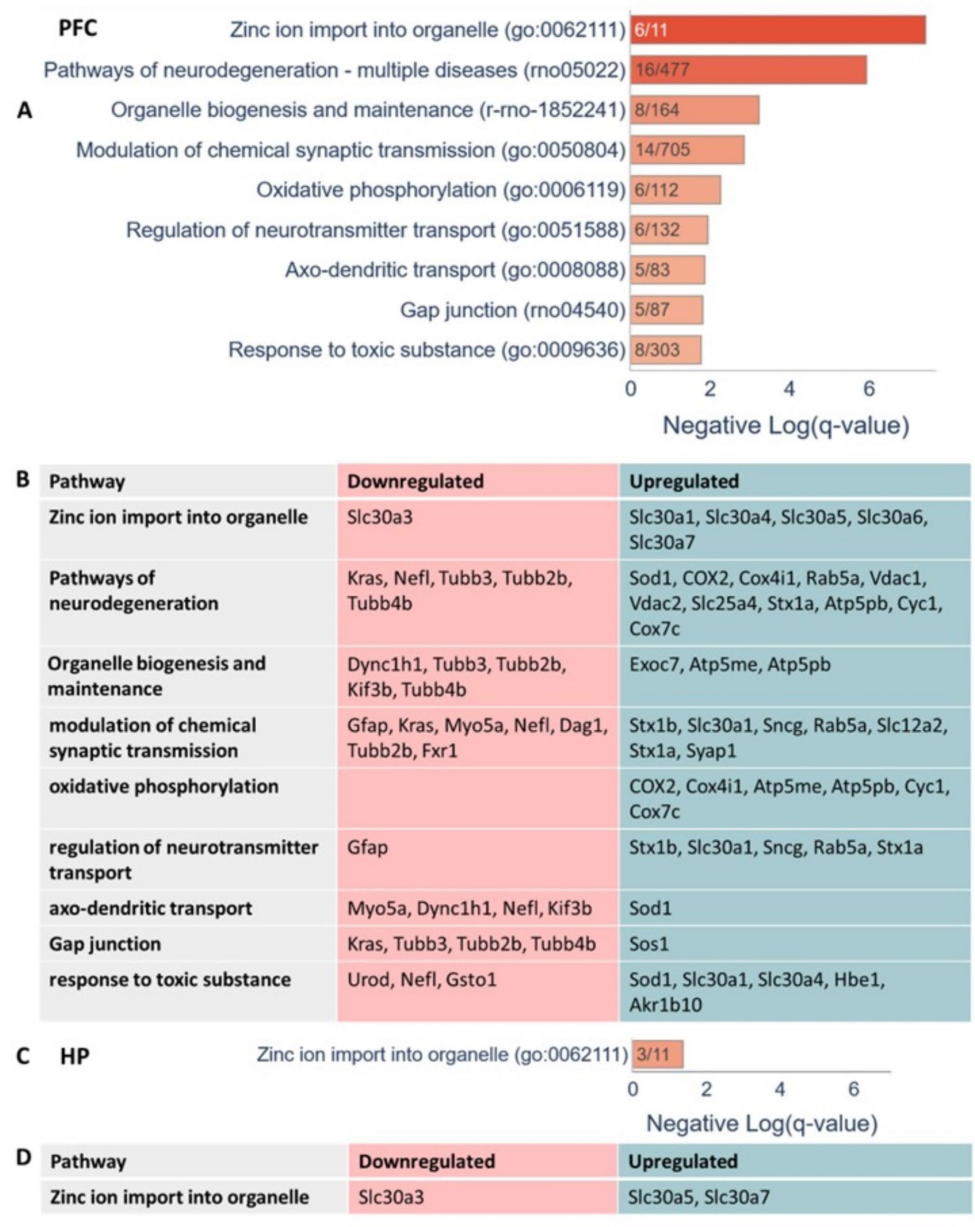
Proteomic pathway enrichment analysis of the impact of zinc-deficient diet (ZnD) on the prefrontal cortex (PFC) and hippocampus (HP) of rats. (**A**) Pathways in the PFC significantly affected by ZnD. The values represent the negative log10-transformed q-values for each pathway, indicating their statistical significance. (**B**) Proteins in the PFC that are either downregulated or upregulated in ZnD-exposed rats, grouped by the pathways identified in Panel A. (**C**) Pathways in the HP significantly affected by ZnD, focusing on zinc ion transport into organelles. The negative log10-transformed q-values indicate the degree of statistical significance for these pathways, which differ from those observed in the PFC. (**D**) Proteins in the HP involved in zinc ion transport pathways identified in Panel C, with changes in expression in ZnD-exposed rats

**Fig. 2:**
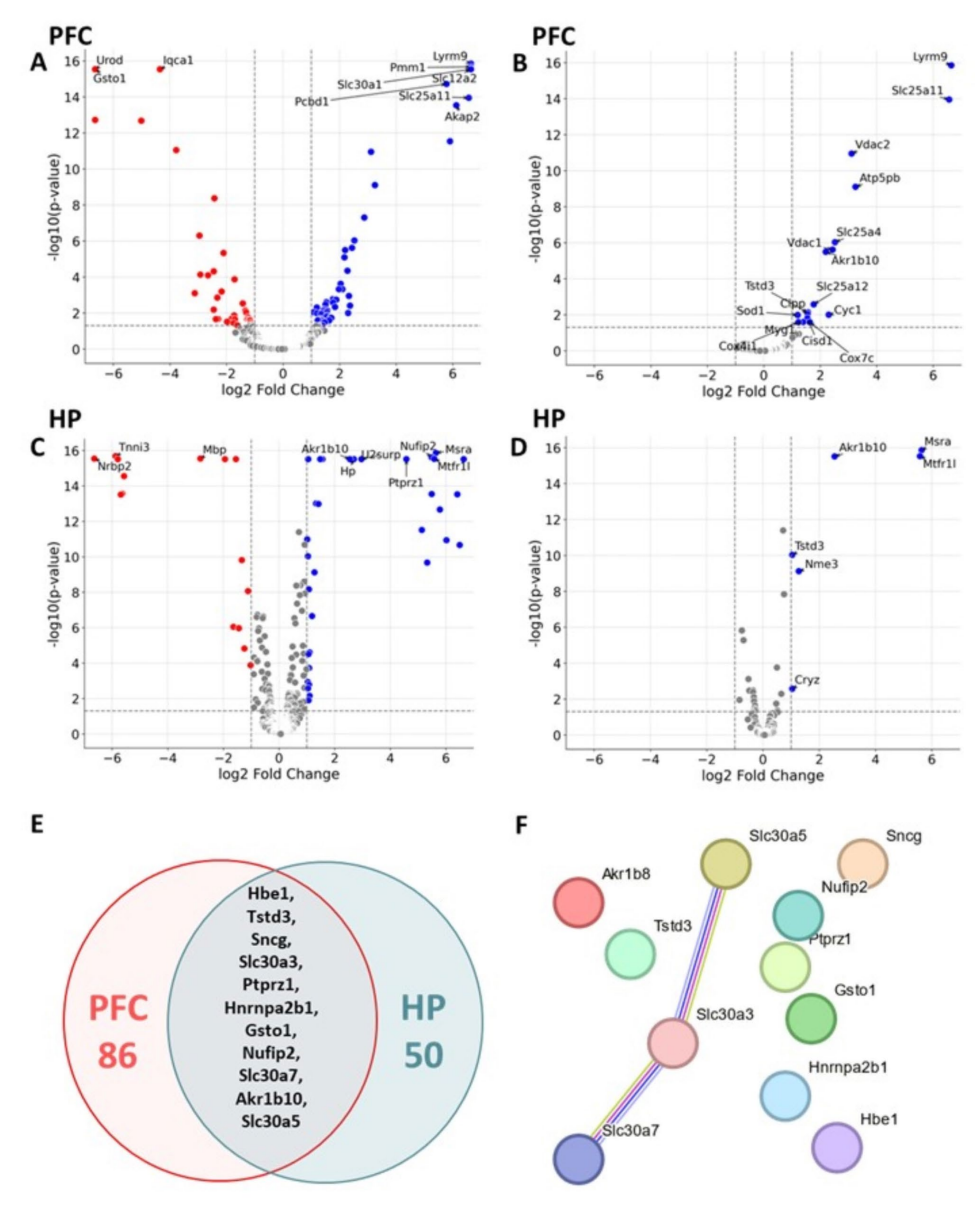
Comparative analysis of zinc-deficient diet (ZnD) effects on proteins in prefrontal cortex (PFC) and hippocampus (HP), including mitochondrial protein identification. (**A**,** B**) Volcano plots for the PFC (**A**) and HP (**C**) display log2 fold changes (x-axis) and negative log10-transformed FDR-adjusted *p*-values (y-axis) for all identified proteins. The top 10 differentially expressed proteins (DEPs) with the most significant changes are highlighted in each plot. Horizontal dashed lines indicate the q-value threshold (0.05, approximated as -log10 ≈ 1.33), while vertical dashed lines represent log2 fold change cutoffs of ± 1. (**A**,** C**) Volcano plots for mitochondrial proteins identified from the Mouse MitoCarta3.0 compendium (1140 genes) show only those detected within the dataset. Panels **B** and **D** depict mitochondrial-specific changes in the PFC and HP, respectively, highlighting statistically significant mitochondrial proteins affected by ZnD. (**E**) A Venn diagram illustrates the overlap of DEPs between the PFC and HP, showing 86 DEPs unique to the PFC, 50 unique to the HP, and 11 shared across both regions. (**F**) STRING network analysis of the 11 shared DEPs visualizes functional interactions among these proteins, including links between zinc transporters (e.g., Slc30a3 and Slc30a7) and other proteins detected in the analysis

Pathway and process enrichment analysis using Metascape revealed that these differentially expressed proteins (DEPs) in the PFC were significantly enriched in pathways related to zinc ion import into organelles (q-value = 4.00e-08) (Fig. 1A). Notably, proteins known to be modulated by zinc levels, such as Scl30a1, Slc30a4, Scl30a5, Scl30a6 and Slc30a7were upregulated, while Slc30a3 was downregulated (Fig. 1B). Among the changed proteins were also proteins involved in neurodegeneration pathways (q-value = 1.00e-06), organelle biogenesis (q-value = 6.00e-04), regulation of neurotransmitter transport (q-value = 0.01), axo-dendritic transport (q-value = 0.014), gap junctions (q-value = 0.016) and response to a toxic substance (q-value = 0.015) (Fig. 1A). The oxidative phosphorylation pathway, which includes mitochondrial proteins such as COX2 and Atp5pb, was notably affected (q-value = 0.015), emphasizing the profound impact of ZnD on energy production in the PFC (Fig. 1B). A comparative analysis of ZnD effects on proteins in PFC, including mitochondrial protein identification, is shown in Fig. 2A, B.

In the HP, proteomic analysis of the post-ZnD revealed significant alterations in 50 proteins out of 2,591 identified, with 35 upregulated and 15 downregulated, as detailed in Supplementary Table S2. These DEPs were significantly involved in zinc ion import into organelles (q-value = 0.043, Fig. 1C), mirroring observations in the PFC and suggesting a systemic zinc transport pathway response across both brain regions. A comparative analysis of ZnD effects on proteins in HP, including mitochondrial protein identification, is shown in Fig. 2C, D.

Notably, a four-week zinc deprivation led to more pronounced changes in the PFC than in the HP, evidenced by a higher number of DEPs and more significant fluctuations in protein levels in the PFC (Figs. 1A-D and 2A and C), suggesting differential regional sensitivity to zinc depletion. However, 20% of the proteins differentially regulated in the HP also showed modulation in the PFC, indicating a shared response mechanism to ZnD within these rat brain regions, as depicted in the Venn diagram (Fig. 2E). The proteins common to both brain regions include Hbe1, Tstd3, Sncg, Slc30a3, Ptpr1, Hnrnpab2b1, Gsto1, Nutfp2, Slc30a7, Akr1b10, and Slc30a5.

The STRING network diagram (Fig. 2F) illustrates the interactions between these common proteins. This analysis reveals how the zinc transporters collaborate in the PFC and HP in zinc-deficient rats.

### ZnD induces alterations in the activities of respiratory complexes I and IV in the PFC of rats

**Fig. 3:**
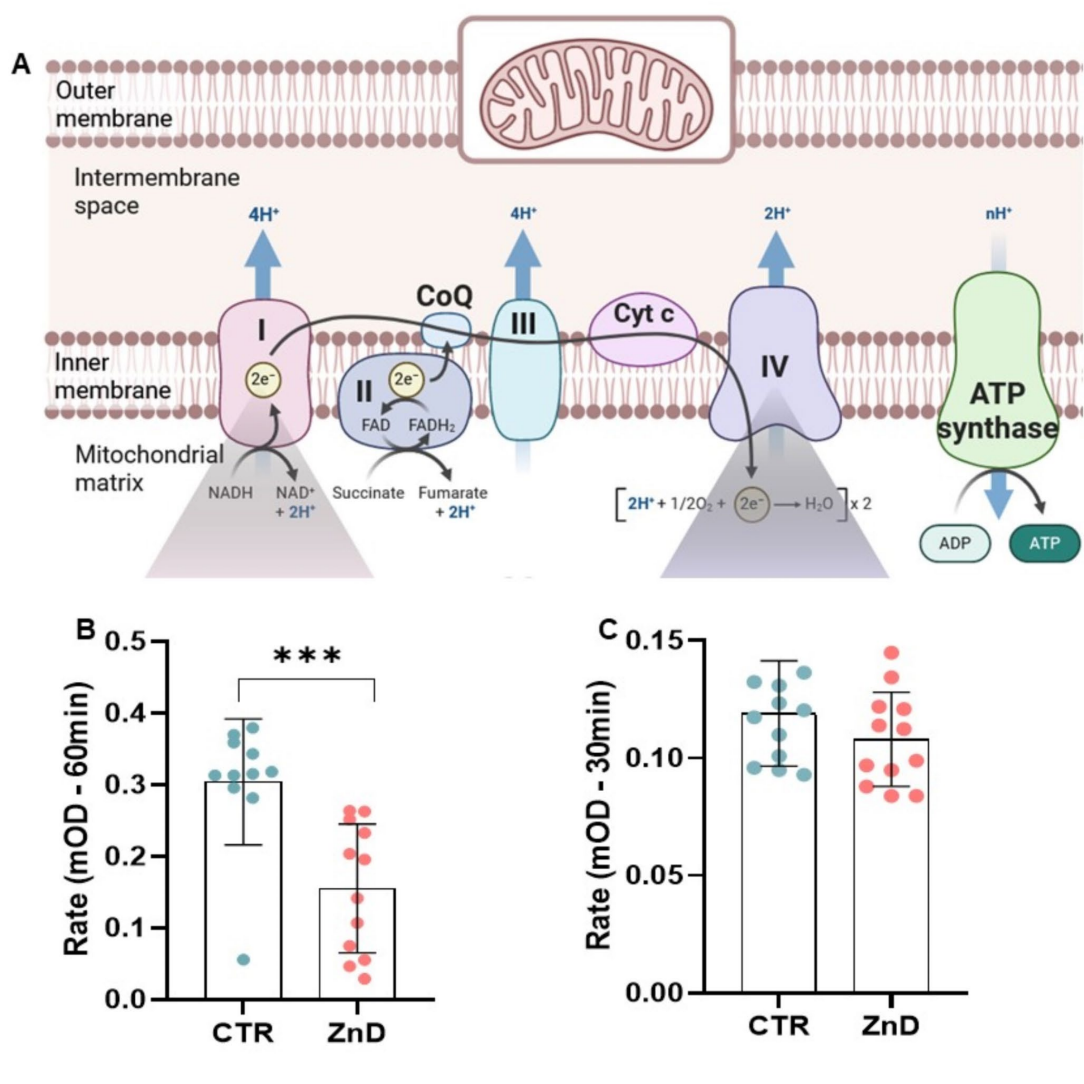
Impact of zinc-deficient diet (ZnD) on mitochondrial respiratory complex activities. (**A**) Schematic representation of the mitochondrial electron transport chain, showing Complexes I-IV and ATP synthase within the inner mitochondrial membrane. Complex I (NADH: ubiquinone oxidoreductase), Complex II (succinate dehydrogenase), Complex III (cytochrome c reductase), Complex IV (cytochrome c oxidase), and ATP synthase are key to oxidative phosphorylation and ATP production. (**B**) Activity of Complex I in CTR and ZnD conditions. Data are presented as the median ± interquartile range (IQR). (**C**) The activity of Complex IV in CTR and ZnD conditions. Data are presented as mean ± standard deviation (SD). ***p = 0.0001. CTR - control, ZnD - zinc deficient diet

Given the observed association of numerous proteins with electron transport in the respiratory chain within the cortex, potentially linked to energy production, and considering previous literature that correlated alterations in the activities of respiratory complexes I and IV with depression [35–37], we opted to validate these proteomic results for the PFC. Since no changes in respiratory chain protein levels were observed in the HP, validation was not performed for this region. We conducted assays using the Complex Enzyme Activity Assay Kit and Complex IV Rodent Enzyme Activity Microplate Assay Kit. Results indicated a significant decrease in Complex I activity in the PFC of rats subjected to the ZnD compared to control (CTR) rats (Mann-Whitney U test, U = 9, *p* = 0.0001, two-tailed; Fig. 3B). The median activity was 0.1685 for ZnD and 0.3150 for CTR, with a Hodges-Lehmann median difference of -0.1473. In contrast, no significant changes were detected in Complex IV activity (unpaired t-test, t_22_ = 1.261, *p* = 0.2204, two-tailed; Fig. 3C). Mean activity for complex IV was 0.1080 in the ZnD group and 0.1190 in CTR, with a mean difference of -0.01096 ± 0.008688 (95% CI: [-0.02898, 0.007059]).

## Discussion

This study provides comprehensive data on the proteome of rat brain structures, focusing on the effects of a ZnD. Our data showed numerous changes in protein levels in PFC and HP after dietary intervention. We found 86 differentially regulated proteins in PFC and 50 in HP compared to the control counterparts. The gene ontology (GO) analysis of deregulated protein sets revealed alterations in pathways of many cellular functions. In general, the deregulated pathways were mainly involved in zinc transport transmembrane activity, oxidative phosphorylation, regulation of neurotransmitter transport, gap junctions, and response to toxic substances. Meanwhile, the corresponding analysis of HP revealed fewer changes, which were mainly related to zinc transport.

The analysis of similarities in the pattern of protein expression between PFC and HP in the zinc-deficient rats revealed eleven proteins: Hbe1, Tstd3, Sncg, Slc30a3, Ptprz1, Hnrnpa2b1, Gsto1, Nufip2, Slc30a7, and Akr1b10. One overlapping protein, Sncg (synuclein-gamma), a member of the synuclein family, is suggested to be involved in neurodegenerative pathology in mice [38] and in the pathophysiology of depression in rat models [39]. Although the zinc-alpha-synuclein interaction has been suggested as the mechanism involved in the pathophysiology of Parkinsons disease (PD) [40], the influence of zinc on gamma-synuclein is not mentioned in the literature. Clinical studies indicated lower levels of zinc in the serum and plasma in patients with PD [41, 42]. Lower serum and plasma zinc levels were also identified as a factor increasing the risk of developing this disease [40]. Therefore, our findings may suggest a new target or mechanism involved in the progression or development of PD and depression induced by zinc deficiency.

A potential link between zinc and depression may involve zinc-finger proteins, critical in regulating gene transcription. Zinc is an essential component of zinc fingers, a structural motif in many transcription factors that regulate genes associated with cell development, differentiation, and stress responses, influencing neuroplasticity and neuronal function. Zinc is also a cofactor in more than 300 enzymes and proteins, underscoring its crucial role in various cellular processes, including DNA synthesis, cell division, energy metabolism, and neurotransmission [43]. In our study, the expression of ZBTB18 (Zinc Finger and BTB domain-containing protein 18) was reduced in the HP (see Supplementary Table S2). ZBTB18 plays a critical role in neuroplasticity and the development of cortical neurons [44]. Research indicates that mutations in ZBTB18 can impair its DNA-binding ability, disrupting the regulation of genes critical for neuronal migration and differentiation [45]. This dysfunction could impact neuroplasticity-related gene expression, potentially connecting zinc deficiency to the depressive phenotype observed in our model.

Another protein deregulated in the brain of rats subjected to ZnD is glial fibrillary acidic protein (GFAP). Multiple studies have found decreased expression levels of GFAP in the cortex to be associated with MDD [46–48]. In line with these findings, it is possible to hypothesize that altered GFAP protein levels may be a common factor linking ZnD and depressive disorder or a mechanism by which ZnD might induce depression.

In mammalian cells, zinc transporters (ZnTs) act as the primary metal proton exchangers responsible for zinc efflux from cells or influx into cellular compartments or organelles [49]. Among the ten known members of the ZnT family [50], we found that five were differentially regulated in PFC after four weeks of dietary intervention (ZnT1, ZnT4, ZnT6, and ZnT7 were upregulated, and ZnT3 downregulated), while in HP, ZnT3 was downregulated and ZnT6, as well as ZnT7, upregulated. These findings suggest a global redistribution of zinc in the nervous system due to zinc deficiency. Interestingly, the mechanisms of zinc redistribution seem to be brain structure-related, showing a general increase in the zinc transporters expression in the PFC with less effect in the HP. When comparing our results to those of older studies, it must be pointed out that the ZnTs expression pattern observed in this experiment corresponds to our previous studies, where we observed high levels of ZnT1, ZnT4, and ZnT5 in the PFC of humans with diagnosed MDD [51]. Among all differentially expressed ZnT transporters, ZnT3 was less expressed in both the PFC and HP after dietary intervention. As ZnT3 is involved in sequestering zinc into synaptic vesicles, it has been extensively studied in PFC and HP, especially for its role in spatial memory and behavioral regulation. Particularly in the HP, ZnT3 has been shown to regulate cell proliferation and neuronal differentiation [50, 52, 53]. Apart from ZnTs, the Zrt-, Irt-like protein (ZIP) family is another crucial group of zinc transporters responsible for zinc flux into cells. Additionally, intracellular zinc is modulated by binding to cysteine-rich metallothioneins (MTs), which buffer and traffic the metal within cells [54]. However, we observed no differences in the ZIP family proteins and metallothioneins, which suggests that ZnTs are the most relevant regulators of the homeostatic response to diminished zinc levels in the diet.

Numerous studies highlight zinc’s modulatory role in regulating glutamate receptors, especially NMDA receptors and GABA receptors [55, 56], as well as its impact on neurotrophins like brain-derived neurotrophic factor (BDNF), which are involved in neuroplasticity [57]. However, our study did not detect any changes in the levels of these receptors or BDNF in the PFC and HP (see Supplementary Table S1). As was mentioned above, we did not observe any alterations in the levels of metallothioneins (MTs), which are crucial zinc-binding proteins involved in oxidative stress protection and zinc homeostasis. In other depression models, such as the chronic mild stress model, changes in MT expression have been observed following treatment with fluoxetine and zinc supplementation [58]. Our findings indicate that zinc’s effects on depression are highly complex, with ZnD and supplementation not exhibiting simple inverse outcomes. Future studies should adopt more targeted hypotheses, focusing on the specific conditions under which zinc modulates distinct pathways and how these effects may differ across various depression models.

Previous studies have emphasized that MDD occurs more frequently in patients with dysregulated metabolism. For instance, depression often coexists with type 2 diabetes [59], metabolic syndrome [60], and glucose metabolism disturbances [61]. Neurons are particularly vulnerable to mitochondrial dysfunction due to their high energy demands. It has been estimated that cortical neurons are highly energy-consuming, demanding 4.7 billion ATP molecules per second compared to the utilization of only 10 million ATP molecules per second in non-neuronal cells of the human brain [62]. Thus, the brain is one of the most energy-consuming organs, using up to 20% of total oxygen metabolism and accompanied by a low available energy reserve [63]. Recent studies have indicated that neurotransmitters’ release, neurogenesis, and decreased neuroplasticity could be coupled with cellular metabolism and mitochondrial abnormalities [64–66]. All of these metabolically dependent processes mentioned above are, at the same time, the main factors involved in MDD pathophysiology [67]. This observation highlights the loss of mitochondrial quality and lack of brain energy homeostasis as the significant factors involved in the development of depressive disorder [64–66].

As previously reported in the literature, maintaining zinc homeostasis is conducive to mitochondrial pyruvate transport, oxidative phosphorylation, tricarboxylic acid (TCA) cycle, carbohydrate metabolism, glycolysis, and global energy metabolism [68–72]. Recent studies have indicated that zinc deficiency has an unambiguously deleterious effect on mitochondrial quality and energy production. For instance, low zinc levels have been associated with increased mitochondrial oxidative stress [73], impairment of mitochondrial biogenesis in the liver [70], and compromised glycolysis in hepatocytes [69]. While most recent studies focus on the effect of increased zinc levels on the brain and related mitochondrial dysfunctions [74], our data demonstrate metabolic abnormalities in the zinc deficiency model. These observations emphasize the crucial role of zinc homeostasis in maintaining neuronal mitochondria quality and adequate brain metabolism.

In our model of depression induced by ZnD, we observed that most dysregulated proteins in PFC and HP are associated with mitochondrial function and ATP production. In the PFC, we observed higher levels of specific proteins involved in oxidative phosphorylation at Atp5pb, Cox2, Atp5me, Cyc1, Cox4i1, Cox7c, and mitoNEET (Cisd1). Most of the differentially regulated proteins involved in mitochondrial electron transport in PFC are the components of the respiratory complex IV (cytochrome c oxidase). This result is in line with previous studies showing dysregulation of mitochondrial proteins mainly involved in respiratory complexes I and IV in a broad spectrum of psychiatric disorders, including schizophrenia, bipolar disorder, and depression [35, 36, 75, 76]. Our present studies indicated that ZnD induced a more pronounced decrease in the activity of complex I than complex IV compared to controls.

Interestingly, in our research, we did not observe changes in the proteins of complex I. However, previous research has demonstrated that various NDUF proteins of complex I are highly dependent on zinc levels. For example, the NDUFS6 subunit harbors a critical zinc-binding site necessary for the assembly and function of complex I [77]. Both an excess and deficiency of zinc have been shown to severely inhibit the activity of complex I, reducing energy production in neurons and potentially leading to cellular death [78, 79].

Our study also indicates altered levels of the critical mitochondrial membrane transporters in the PFC. Notably, among the zinc transporters, ZnT7 deserves special attention in the context of mitochondrial dysfunction. Our data demonstrate increased ZnT7 levels in the PFC and HP of the ZnD rats. Studies by Tuncay et al. well documented ZnT7 as a significant zinc transporter into both the endoplasmic reticulum and mitochondria, showing the correlation between increased ZnT7 levels and elevated mitochondrial zinc content, reactive oxygen species (ROS) production, and depolarized mitochondrial membrane potential in cardiomyocytes [80].

This evidence indicates that the pro-depressive effects of ZnD may be primarily attributed to mitochondrial dysfunction, particularly affecting oxidative phosphorylation and ROS production, which could contribute to neuronal dysfunction and the observed behavioral deficits.

The proteomic analysis also indicates that the PFC of zinc-deficient rats exhibits a high expression of two voltage-dependent anion-selective channel proteins, VDAC1 and VDAC2. The VDACs are the essential proteins regulating metabolic and energetic flux across the outer mitochondrial membrane by transporting ATP, ADP, pyruvate, malate, Ca2+, and other metabolites [81]. Therefore, VDACs, as mitochondrial porins, play a pivotal role in maintaining the metabolic crosstalk between the mitochondria and cytoplasm, ensuring up to 90% of the mitochondrial membrane permeability, thus responsible for proper ATP production, calcium homeostasis, and regulation of apoptosis [82, 83]. In recent years, VDACs have been intensively studied in the context of neurodegenerative disorders like Alzheimer’s disease and Parkinson’s Disease or dementia with Lewy bodies [84, 85]. Interestingly, recent studies have shown that fluoxetine, a commonly used antidepressant, can partially block VDAC [86]. Interestingly, in our previous studies, fluoxetine was demonstrated to reverse depression-like behavior in zinc-deficient rats [20]. Such observations emphasize that VDAC alterations could be another prominent direction for studies linking MDD cases with ZnD.

## Conclusion

Our data indicated that ZnD significantly alters rat PFC and HP protein levels. However, PFC showed more extensive changes than HP. Deregulated pathways mainly involved zinc transport, oxidative phosphorylation, neurotransmitter transport, gap junctions, and mitochondrial function. Previous proteomic studies stress the global metabolic changes and mitochondrial dysfunctions together with cytoskeleton alterations as the most common features linking the various models of depression. Zinc deficiency-induced impairment of mitochondrial function might be a possible mechanism linking it with neurodegenerative diseases and depression. As mitochondria are indispensable for sustaining the high energy demand of neurons, an in-depth understanding of mitochondria in depression-associated mechanisms is crucial for developing new therapeutic strategies.

Furthermore, ZnD affects the ZnT transporter’s level. Further study on the role of different ZnTs, especially ZnT7, might lead to the development of interventions targeting ZnTs to restore zinc levels, mitigate mitochondrial dysfunction in maintaining zinc homeostasis, and prevent depressive symptoms. Our data also showed that ZnD influences the Sncg protein level. Exploring the zinc-sigma-synuclein interaction might help to explain the contribution of ZnD to neurodegenerative diseases and find treatment strategies.

We present changes resulting from a zinc-deficient diet over four weeks. It would be interesting to investigate whether these changes persist after a more extended period of zinc deficiency or if compensatory mechanisms are triggered. Additionally, it would be valuable to confirm whether similar proteomic changes occur in individuals with dietary zinc deficiency and depression.

## Electronic supplementary material

Below is the link to the electronic supplementary material.

Supplementary Material 1

Supplementary Material 2

## Data Availability

No datasets were generated or analysed during the current study.
